# Brazilian Academy of Neurology practice guidelines for stroke rehabilitation: part I

**DOI:** 10.1590/0004-282X-ANP-2021-0354

**Published:** 2022-08-08

**Authors:** Cesar Minelli, Rodrigo Bazan, Marco Túlio Araújo Pedatella, Luciana de Oliveira Neves, Roberta de Oliveira Cacho, Sheila Cristina Sayuri Abe Magalhães, Gustavo José Luvizutto, Carla Heloísa Cabral Moro, Marcos Christiano Lange, Gabriel Pinheiro Modolo, Bruna Correia Lopes, Elisandra Leites Pinheiro, Juli Thomaz de Souza, Guilherme Riccioppo Rodrigues, Soraia Ramos Cabette Fabio, Gilmar Fernandes do Prado, Karla Carlos, Juliana Junqueira Marques Teixeira, Clara Monteiro Antunes Barreira, Rodrigo de Souza Castro, Thalita Dayrell Leite Quinan, Eduardo Damasceno, Kelson James Almeida, Octávio Marques Pontes-Neto, Marina Teixeira Ramalho Pereira Dalio, Millene Rodrigues Camilo, Michelle Hyczy de Siqueira Tosin, Bianca Campos Oliveira, Beatriz Guitton Renaud Baptista de Oliveira, João José Freitas de Carvalho, Sheila Cristina Ouriques Martins

**Affiliations:** 1 Hospital Carlos Fernando Malzoni, Matão SP, Brazil.; 2 Universidade de São Paulo, Faculdade de Medicina de Ribeirão Preto, Departamento de Neurociências e Ciências do Comportamento, Ribeirão Preto SP, Brazil.; 3 Universidade Estadual Paulista, Faculdade de Medicina de Botucatu, Botucatu SP, Brazil.; 4 Hospital Israelita Albert Einstein, Unidade Goiânia, Goiânia GO, Brazil.; 5 Hospital Santa Helena, Goiânia GO, Brazil.; 6 Hospital Encore, Goiânia GO, Brazil.; 7 Hospital Geral de Goiânia, Goiania GO, Brazil.; 8 Hospital de Urgência de Goiânia, Goiânia GO, Brazil.; 9 Universidade de Fortaleza, Hospital São Carlos, Fortaleza CE, Brazil.; 10 Universidade Federal do Rio Grande do Norte, Faculdade de Ciências da Saúde do Trairi, Santa Cruz RN, Brazil.; 11 Universidade Federal do Triângulo Mineiro, Departamento de Fisioterapia Aplicada, Uberaba MG, Brazil.; 12 Neurológica Joinville, Joinville SC, Brazil.; 13 Hospital Municipal de Joinville, Joinville SC, Brazil.; 14 Associação Brasil AVC, Joinville SC, Brazil.; 15 Universidade Federal do Paraná, Complexo Hospital de Clínicas, Curitiba PR, Brazil.; 16 Hospital Moinhos de Vento, Porto Alegre RS, Brazil.; 17 Hospital UNIMED Ribeirão Preto, Ribeirão Preto SP, Brazil.; 18 Universidade Federal de São Paulo, Escola Paulista de Medicina, São Paulo SP, Brazil.; 19 Hospital Orion, Goiania GO, Brazil.; 20 Universidade Federal do Piauí, Departamento de Neurologia, Teresina PI, Brazil.; 21 Universidade de São Paulo, Hospital das Clínicas, Faculdade de Medicina de Ribeirão Preto, Centro de Cirurgia de Epilepsia de Ribeirão Preto, Ribeirão Preto SP, Brazil.; 22 Universidade Federal Fluminense, Niterói RJ, Brazil; 23 Rush University, Chicago IL, USA; 24 Faculdade de Medicina Unichristus, Fortaleza CE, Brazil.; 25 Hospital Geral de Fortaleza, Fortaleza CE, Brazil.; 26 Rede Brasil AVC, Porto Alegre RS, Brazil.; 27 Hospital Moinhos de Vento, Departamento de Neurologia, Porto Alegre RS, Brazil.; 28 Hospital de Clínicas de Porto Alegre, Departamento de Neurologia, Porto Alegre RS, Brazil.

**Keywords:** Stroke, Guideline, Neurological Rehabilitation, Practice Guidelines as Topic, Acidente Vascular Cerebral, Guia, Reabilitação Neurológica, Guias de Prática Clínica como Assunto

## Abstract

The Guidelines for Stroke Rehabilitation are the result of a joint effort by the Scientific Department of Neurological Rehabilitation of the Brazilian Academy of Neurology aiming to guide professionals involved in the rehabilitation process to reduce functional disability and increase individual autonomy. Members of the group participated in web discussion forums with predefined themes, followed by videoconference meetings in which issues were discussed, leading to a consensus. These guidelines, divided into two parts, focus on the implications of recent clinical trials, systematic reviews, and meta-analyses in stroke rehabilitation literature. The main objective was to guide physicians, physiotherapists, speech therapists, occupational therapists, nurses, nutritionists, and other professionals involved in post-stroke care. Recommendations and levels of evidence were adapted according to the currently available literature. Part I discusses topics on rehabilitation in the acute phase, as well as prevention and management of frequent conditions and comorbidities after stroke.

## RESUMO

As Diretrizes Brasileiras para Reabilitação do AVC são fruto de um esforço conjunto do Departamento Científico de Reabilitação Neurológica da Academia Brasileira de Neurologia com o objetivo de orientar os profissionais envolvidos no processo de reabilitação para a redução da incapacidade funcional e aumento da autonomia dos indivíduos. Membros do grupo acima participaram de fóruns de discussão na web com pré-temas, seguidos de reuniões por videoconferência em que as controvérsias foram discutidas, levando a um consenso. Essas diretrizes, divididas em duas partes, focam as implicações de recentes ensaios clínicos, revisões sistemáticas e metanálises sobre reabilitação do AVC. O objetivo principal é servir de orientação a médicos, fisioterapeutas, fonoaudiólogos, terapeutas ocupacionais, enfermeiros, nutricionistas e demais profissionais envolvidos no cuidado pós-AVC. As recomendações e níveis de evidência foram adaptados de acordo com a literatura disponível atualmente. Aqui é apresentada a Parte I sobre tópicos de reabilitação na fase aguda, prevenção e tratamento de doenças e comorbidades frequentes após o AVC.

## INTRODUCTION

Stroke is the second leading cause of death and disability worldwide^1^ ^,^ ^2^. It is estimated that in 2016, there were almost 260,000 stroke cases, approximately 107,000 deaths, and more than 2.2 million adjusted life years lost due to disability following a stroke in Brazil^3^ ^,^ ^4^. Worldwide, stroke is the most prevalent neurological disease that needs rehabilitation, with 86 million disabled individuals^5^. More than two-thirds of individuals after stroke receive rehabilitation services after hospitalization^6^. Despite the development and support of stroke centers and national societies in Brazil to raise awareness of stroke symptoms, only a minority of stroke patients in the acute phase receive thrombolytic therapy or thrombectomy. Consequently, many stroke survivors have residual functional deficits. Stroke rehabilitation differs in many regions in Brazil according to socio-economic conditions. In large urban centers stroke patients are referred to a rehabilitation center by the time of discharge; however, in most parts of the country stroke survivors have few opportunities to initiate or continue rehabilitation after the acute phase. This data is lacking in Brazil and has been evaluated by the Access to Rehabilitation Study across 17 public health centers in Brazilian cities in the North, Northeast, West, Southeast and South of Brazil^7^. Therefore, the need for effective rehabilitation of stroke patients remains an essential part of the continuum of stroke treatment.

Considering this premise, the Scientific Department of Neurological Rehabilitation of the Brazilian Academy of Neurology made efforts to draft the first Brazilian Guidelines for Stroke Rehabilitation to guide professionals involved in the rehabilitation process to reduce functional disability and increase the autonomy of individuals. The members of the group participated in discussion forums on the web with predefined themes, followed by videoconference meetings in which controversies were discussed, leading to a consensus. For the preparation of the Brazilian Guidelines for Stroke Rehabilitation, several national co-authors, with prior knowledge in their areas of expertise, were asked to write the suggested topics following criteria defined by the coordinators of these guidelines. The original texts were adapted to follow a format in which, after the general information, the recommendations for each intervention were added. 

The present work focuses on recent clinical trials, meta-analyses, and systematic reviews in stroke rehabilitation literature. The main objective of this paper is to guide physicians, physiotherapists, speech therapists, occupational therapists, nurses, nutritionists, and other professionals involved in post-stroke care. Recommendations and levels of evidence have been adapted according to currently available literature. 

We have sought to provide visibility to broader rehabilitation aspects based on the intervention concepts proposed in the International Classification of Functioning, Disability, and Health^8^. The rehabilitation strategies included in this guideline cover the different stroke phases: hyper-acute (0-24 hours), acute (1-7 days), early subacute (7 days-3 months), late subacute (3-6 months), and chronic phases (> 6 months)^9^. Most studies in stroke rehabilitation include participants over the age of 18 years^10^. In clinical practice, the same interventions are used for all ages. Therefore, the recommendations of this guideline can be applied to all individuals after a stroke. In addition, these guidelines also aim to highlight the issues of accessibility and palliative care. The guidelines have been divided into two groups. Part I includes topics on rehabilitation in the acute phase as well as prevention and management of the most frequent conditions and comorbidities after stroke. A section on Secondary Stroke Prevention was included in Part I because the incidence of stroke recurrence is higher in the first months after stroke^9^ and it is a potential and preventable complication that impairs the process of rehabilitation as do falls, deep vein thrombosis and others. More detailed information about Secondary Stroke Prevention is available at https://www.ahajournals.org/doi/pdf/10.1161/STR.0000000000000375. Table 1 shows daily doses, adverse effects, and duration of follow-up during the study periods of drugs used in the management of central pain, mood disorder, sleep disorder, and epilepsy after stroke. Part II covers the topics on rehabilitation of neurological deficits and disabilities after stroke, and transitions to community rehabilitation and palliative care. A table with validated scales to assess neurological impairment, disability, and quality of life is included in Part II. At the end of Part II, supporting material includes suggestions for patients, caregivers, and other health professionals, including legal rights after stroke, as well as functional accessibility laws and the care network. We have also included a chapter on the possibilities of paths to be followed in the future, based on promising approaches to rehabilitation after stroke.

**Table 1.** Daily doses, adverse effects, and duration of follow-up during the study periods of drugs used in the management of central pain, mood disorder, sleep disorder, and epilepsy after stroke.

Comorbidities	Drug	Daily dose	Duration of study follow-up	Adverse effects
Central Pain	Amitriptyline^39^	Start with 25 mg, up to 75 mg	4 weeks	Dry mouth, urinary retention, drowsiness, and confusion
	Lamotrigine^39^	Start with 25 mg, up to 200 mg	8 weeks	Rash and severe headache
	Duloxetine^42^	Start with 30 mg, up to 60 mg	3 weeks	Nausea, agitation, and drowsiness
	Pregabalin^43^	Start with 150 mg, up to 600 mg	12 weeks	Somnolence, and peripheral edema
	Gabapentin^39^	Start with 900 mg, up to 2400 mg	8 weeks	Dizziness and drowsiness
	Fluvoxamine^44^	Start with 50 mg, up to 125 mg	2 to 4 weeks	Drowsiness, insomnia, and restlessness
Mood disorder	Nortriptyline^79^	Start with 25 mg, up to 100 mg	6 weeks	Dry mouth, urinary retention, drowsiness, and confusion
	Trazodone^79^	Start with 25 mg, up to 200 mg	32 days	Dry mouth, urinary retention, drowsiness, and confusion
	Citalopram^79^	Start with 5 mg, up to 10 mg	6 weeks	Nausea, drowsiness, weakness, dizziness, anxiety, trouble sleeping, and sexual dysfunction
	Fluoxetine^79^	Start with 10 mg, up to 10 mg	6 weeks to 3 months	Nausea headaches, insomnia, diarrhea, weakness, and anxiety
	Reboxetine^79^	4 mg	16 weeks	Dry mouth, constipation, and sexual dysfunction
Sleep disorders	Trazodone^110^	100 mg	1 week	Dry mouth, urinary retention, somnolence, and confusion
Epilepsy	Levetiracetam^157^	Start with 500 mg up to 300 mg	52 weeks	Fatigue, drowsiness, skin eruptions or allergies
	Lamotrigine^157^	Start with 25 mg up to 200 mg	52 weeks	Dizziness and rash
	Controlled release carbamazepine^157^	Start with 200 mg up to 1600 mg	52 weeks	Confusion, skin eruptions or allergies, nausea, and vomiting

We hope that this pioneering Brazilian work will soon be followed by new versions that can improve and update the content presented here. 

## RECOMMENDATION RATING AND LEVEL OF EVIDENCE

The recommendation rating and level of evidence used in these guidelines is an adaptation of the framework established by the American Heart Association^10^.

### Recommendations

Class I: There is evidence and/or consensus that intervention is effective.

Class II: There is conflicting evidence and/or divergence of opinions about the effectiveness and usefulness of intervention.

a) Although there is divergent evidence on the usefulness and effectiveness of intervention, the recommendations are in favor of intervention;

b) Utility and effectiveness are less established by the evidence or opinions.

Class III: There is evidence and/or consensus that intervention is not useful or effective and may cause harm.

### Levels of evidence

A: Data are obtained from multiple randomized clinical trials or meta-analyses.

B: Data are obtained from a single randomized or non-randomized study.

C: Consensus and expert opinion, case studies, or usual (standardized) treatments.

## ORGANIZATION OF POST-STROKE REHABILITATION CARE (LEVELS OF CARE) 

The ideal organization of post-stroke rehabilitation care includes rehabilitation during the acute phase in stroke units, nursing home facilities, inpatient, home-based and outpatient rehabilitation services^11^. The level of care to which patients will be referred depends on the status of clinical conditions and the degree of neurological impairment and disability. These services should be delivered by a multidisciplinary team with physicians, physical, occupational, speech and language therapists, physical educators, social workers, psychologists, and psychiatrists^10^. Integration within the whole system of health and social community care is necessary. At all levels of care, specific needs should be assessed, such as swallowing, hydration and nutrition, continence, mobility, activities of everyday life, communication, cognition, alertness and engagement, vision, hearing, perception, behavior, emotional, need for assistance, and social engagement^11^. 

The level of care after stabilization of the acute phase will depend on the degree of dependence in activities of daily living, status of comorbidities and neurological impairments and disabilities. It is suggested that the Assessment for Rehabilitation Tool (ART)^12^, a pathway and decision tool that considers individual particularities, such as age, prognosis, neurological impairment and disability domains, level of function, and management level available, i.e., inpatient, home or outpatient rehabilitation. ART also considers exceptions where there is no need to initiate rehabilitation, such as the patient returning to pre-morbid function, coma and/or unresponsiveness or palliative care. 

### Recommendation

1. Organized, coordinated, and multidisciplinary care should be available to patients after stroke. (Recommendation I-A).

## REHABILITATION IN THE ACUTE PHASE

This topic will address themes of relevance to rehabilitation in the acute phase of stroke that do not involve reperfusion or clinical stabilization interventions, as there are specific guidelines for that purpose^13^ ^,^ ^14^.

All patients must be evaluated by a multidisciplinary team using an objective framework, through the application of scales to assess the risk of pulmonary aspiration, malnutrition, pressure ulcers, deep vein thrombosis, neurological deficits, focal and global disabilities, and psychiatric disorders ^9^. A multidisciplinary team should include physicians, physical, occupational, speech and language therapists, physical educators, social workers, psychologists, and psychiatrists^11^. 

All rehabilitative interventions should be initiated as soon as the impairments and disabilities after stroke are diagnosed and should be continued as outpatient rehabilitation in the community^11^ ^,^ ^15^. Some conditions are contraindications to the commencement of rehabilitation: early deterioration, immediate surgery, another serious medical illness or unstable coronary condition, systolic blood pressure lower than 110 mm Hg or higher than 220 mm Hg, oxygen saturation lower than 92% with oxygen supplementation, resting heart rate of less than 40 beats per min or more than 110 beats per min, and temperature higher than 38·5°C^16^. Mobilization out of bed or any other intervention should be initiated only if the patient’s blood pressure does not drop by more than 30 mm Hg on achievement of an upright position^16^.

Regarding mobilization in the acute phase, the AVERT^16^ multicenter trial showed that the group that received very early mobilization, within 24 hours of stroke onset, had a lower chance of favorable results at three months. Mobilization to maintain range of motion, sensory stimulation and body posture change is not considered intensive rehabilitation^16^. A multicenter study (HeadPoST)^17^ did not find differences between outcomes when comparing a group that rested with the head in the horizontal position, without elevation (i.e., 0º) in the first 24 hours post-randomization and another group in which the head was elevated to at least 30º. 

## COMPREHENSIVE STROKE CENTER

The Comprehensive Stroke Center (CSC), a combined and integrated service for acute-phase care and rehabilitation, offers the best outcomes^18^. Care in a CSC reduces deaths by two and dependence by six in every 100 patients and promotes the return home of six individuals^18^. It is a cost-effective intervention^15^ ^,^ ^18^ ^,^ ^19^. The benefits of CSCs apply to all stroke cases, regardless of severity, age, sex, and whether the stroke is ischemic, hemorrhagic, or a transient ischemic attack^18^.

Despite the limited number of CSCs in Brazil, patients must be admitted to these units in the acute phase, preferably within the first hours of the stroke^20^. A suspicious case evaluated in a service not dedicated to stroke must be immediately transferred to the nearest qualified unit. All services must offer protocols for managing fever, blood pressure, blood glucose, and dysphagia^20^. Additionally, patients must have their rehabilitation needs assessed within 24-48 hours of admission by members of a multidisciplinary team^11^ ^,^ ^15^. 

There is evidence that individuals with mild stroke may have impairments neglected by professionals in multidisciplinary teams^21^. On the other hand, severely affected patients are not referred to rehabilitation services^22^ . To avoid these situations the ART^12^ can be used to provide an appropriate course of post-stroke rehabilitation. 

### Recommendations

1. All patients in the acute stroke phase must be admitted to specialized stroke care units where they can receive care from a multidisciplinary team. (Recommendation I-A);
2. All patients in the acute stroke phase must be seen by specialized professionals and objectively assessed, with the use of scales, for risk of pulmonary aspiration, malnutrition, pressure ulcers, deep vein thrombosis, neurological deficits, focal and global disabilities, and psychiatric disorders. (Recommendation I-A);
3. Very early and high-intensity mobilization within 24 hours of stroke onset is not recommended. (Recommendation III-A);
4. Keeping the head in the horizontal position, without elevation, did not show benefit in the acute post-stroke phase. (Recommendation III-A).

## CONTRACTURES

Contractures are defined as the shortening or stiffening of muscles, skin, or connective tissue resulting in decreased movement and range of motion^23^. Observational studies have shown the incidence of contractures to be between 15% and 60%, mainly in patients with greater motor impairment^24^. The predictors of contractures include spasticity, muscle weakness, upper limb dysfunction, impaired dexterity, and pain^23^. 

Few studies have addressed the treatment of contractures after stroke. Systematic reviews and randomized studies evaluating passive movement and positioning with limb resting orthoses have shown little evidence of benefits in prevention and treatment of contracture^25^ ^-^ ^29^. A dynamic, progressive orthosis fixed in the forearm to lengthen the wrist in extension in post-stroke hemiplegic patients improved the range of motion and resistance to passive movement, but this benefit was not sustained^30^. A recent meta-analysis of several neurological conditions, including those found in post-stroke patients, for interventions to reduce muscle contractures, did not find convincing evidence in favor of non-surgical interventions, such as stretching, botulinum toxin, electrical stimulation, physical activity, and robot-assisted therapies^31^. Surgical release of the brachial, brachioradialis, and biceps muscles improved pain, passive range of motion, and decreased spasticity of the elbow with a contracture^32^.

### Recommendations

1. Progressive casting and adjustable orthotics may be considered to reduce mild to moderate contractures of the elbow joints. (Recommendation IIb-B);
2. Resting ankle and wrist orthotics may be used to prevent contractures. (Recommendation IIb-B);
3. The effects of stretching, botulinum toxin, electrical stimulation, physical activity, and robot-assisted therapies have not been well established. (Recommendation IIb-B);
4. Surgical interventions in the brachial, brachioradialis, and biceps muscles in elbow contractures might be considered. (Recommendation IIb-B).

## PHYSICAL DECONDITIONING 

People who have had a stroke spend 81% of the day in sedentary time, increasing the risk of glucose intolerance, diabetes, heart disease, mood disorders, cognitive decline, decreased muscle mass, increased dependency for daily activities, stroke recurrence, and death. Physical activity (PA) plays a central role in reducing these risks and improving cardiovascular performance^33^. PA also has benefits for bone structure, fatigue, cognition, mood, wellness, sensation, gait speed, social isolation, and has the potential to reduce treatment costs^34^. 

The recommendations below are based on the American and Canadian guidelines^33^ ^,^ ^35^. 

### Recommendations

1. It is recommended that all post-stroke individuals participate in PA interventions once they are clinically stable. (Recommendation I-A);
2. Assessment of PA must be performed by qualified professionals. (Recommendation I-B);
3. Monitoring of heart rate, blood pressure, and rating of perceived exertion before, during, and after completion of the test is recommended. Cardiac monitoring is recommended if stress testing is performed. (Recommendation I-A);
4. Aerobic training is recommended in a rehabilitation program with the addition of muscle strengthening, task-oriented activities of motor control, balance, gait, and functional use of the upper limb. (Recommendation I-C);
5. It is recommended that a PA program be developed and supervised by physical therapists or cardiovascular rehabilitation specialists. (Recommendation I-C);
6. Exercises to activate a large group of muscles for a sufficient period to produce aerobic effort are recommended. (Recommendation I-B);
7. A minimum period of eight weeks is recommended to obtain significant effects, followed by PA being maintained indefinitely. (Recommendation I-B);
8. A frequency of three times a week of PA and lighter physical activities on other days is recommended. (Recommendation I-B);
9. Sessions lasting more than 20 minutes are recommended, with a period of five minutes of warm-up and relaxation before and after each session. (Recommendation I-B);
10. It is recommended that exercise intensity has individualized parameter values based on the percentage of heart rate reserve, percentage of maximum heart rate, and individual perceived exertion. (Recommendation I-B);
11. It is recommended that the effects of PA be monitored by measures of cardiovascular capacity, blood pressure, lipid profile, fasting blood glucose, waist circumference, medication adherence, tobacco use, cognition, mood, and sleep quality. (Recommendation I-B);
12. A PA program is recommended to be continued by the patient so that he/she can practice on their own. (Recommendation I-B);
13. Clinical dates and stress tests with sub-maximal limits of tolerance should be used for prior evaluation of PA as a reference. (Recommendation IIa-C).

## CENTRAL PAIN

Central pain after stroke is defined as neuropathic pain resulting from spinothalamic or thalamocortical tract lesions in the central nervous system (CNS), affecting patients in the acute or chronic phase after a stroke^36^. As a diagnostic criterion, it is necessary that the pain that occurs after a stroke should be located in a body area corresponding to the CNS lesion and not caused by peripheral neuropathic pain or nociceptive stimuli^37^. Numbness, tingling, or needling sensations may also be present. The onset of symptoms is always gradual, coinciding with improvement in sensory perception and the onset of dysesthesia^38^. The pain can be intermittent or constant and can manifest as hyperalgesia or allodynia^36^. 

Amitriptyline and lamotrigine can be first-line pharmacological treatments^39^ ^-^ ^41^. Duloxetine, as an adjuvant treatment, has shown positive effects in pain reduction^42^. Pregabalin and gabapentin can be considered second-line medications, and pregabalin has a favorable secondary effect of reducing anxiety and improving sleep^43^. Fluvoxamine reduced pain in an open observational study^44^. Levetiracetam and carbamazepine do not improve post-stroke neuropathic pain symptoms^39^. There is no evidence for the use of opioids in the treatment of central post-stroke pain^45^. Table 1 shows the drugs with favorable outcomes. 

Steroids, intravenous infusions of lidocaine, ketamine propofol, repetitive transcranial magnetic stimulation (rTMS), deep brain stimulation, and spinal electrical stimulation showed favorable results^46^ ^,^ ^47^. However, they should be reserved for refractory cases^48^. 

### Recommendations

1. Amitriptyline and lamotrigine should be used as first-line treatments for neuropathic pain. (Recommendation I-A);
2. Duloxetine can be considered as an adjuvant treatment. (Recommendation IIa-B);
3. Pregabalin and gabapentin can be used as second-line medication. (Recommendation IIa-B);
4. Fluvoxamine can be considered. (Recommendation IIb-B);
5. rTMS, deep brain, or spinal electrical stimulation may be considered in refractory cases. (Recommendation IIb-B);
6. Levetiracetam, carbamazepine, and opioids are not recommended. (Recommendation III-B).

## PAINFUL SHOULDER 

Painful shoulder (PS) after stroke has an incidence range of 9% to 73%, depending on the diagnostic criteria used in the studies^49^. It can appear in the first two weeks but is more frequent between the second and fourth months after stroke^50^. The most frequent causes are spasticity, adhesive capsulitis, and glenohumeral subluxation^49^. 

Evidence for the use of shoulder orthoses to prevent dislocation, decrease pain, and improve function is conflicting^50^ ^,^ ^51^. These orthoses can improve gait efficiency^51^. Placing an orthosis on an already dislocated shoulder can reduce vertical subluxation on imaging examinations, but the improvement is not maintained after removing the orthosis^52^. 

Gentle joint alignment movements and mobilization with external rotation and abduction may be beneficial^49^. Analgesics, such as acetaminophen and ibuprofen, and neuromodulators can be used^49^. Botulinum toxin has positive effects on pain reduction and functional improvement and increases the range of motion^53^. Subacromial corticosteroid injections can be used if the pain is caused by trauma or inflammation of the subacromial region^54^. Suprascapular nerve blocks, with and without corticosteroids, increased passive range of motion^55^. A functional bandage reduced shoulder subluxation, improved upper limb motor function and activities of everyday life, and reduced pain when compared to placebo^56^ ^,^ ^57^. 

Conventional acupuncture and electroacupuncture have shown uncertain benefits^58^. Electrical functional stimulation can be beneficial in reducing pain and regaining independence in activities of everyday life^59^. The pulley system should not be used^60^. 

### Recommendations

1. Functional bandages are recommended for PS after stroke. (Recommendation I-A);
2. Botulinum toxin injection in the subscapular and pectoral muscles is recommended, mainly if PS is associated with spasticity. (Recommendation I-A);
3. Arm position and support during rest, arm protection, and support during functional movements can be considered to prevent PS. (Recommendation IIa-C);
4. Functional electrical stimulation can be considered in the prevention of PS. (Recommendation IIa-A);
5. PS can be treated with gentle alignment movements and mobilization with external rotation and abduction. (Recommendation IIa-B);
6. Analgesics, such as acetaminophen and ibuprofen, and neuromodulators, can be used. (Recommendation IIa-A);
7. Subacromial corticosteroid injections and suprascapular nerve block are reasonable options for hemiplegic PS. (Recommendation IIb-B);
8. Acupuncture, as an adjunctive treatment, has an uncertain value. (Recommendation IIb-B);
9. The use of orthotics to prevent dislocations is uncertain. (Recommendation IIb-B);
10. The pulley system should not be used for the prevention of PS. (Recommendation III-A).

## PRESSURE INJURY 

Pressure injury (PI) is defined as localized injury to the skin and/or underlying tissues, usually over a bony prominence, resulting from pressure or pressure in combination with shear^61^. Its etiology is multifactorial and can include advanced age, cognitive, physical, and sensory impairment, comorbid conditions, malnutrition, and limited mobility. 

The PI classification of the Associação Brasileira de Estomatoterapia (SOBEST) and Associação Brasileira de Enfermagem em Dermatologia (SOBENDE) is recomended in these guidelines^62^. 

Although not specific to patients after stroke, the Braden Scale is a widely used tool for assessing pressure injury risk and had moderate predictive validity^63^. The Sunderland Scale and the Cubbin & Jackson Revised Scale can also be used and have been translated and validated in Portuguese^64^. 

 The recommendations for the prevention and care of PI after stroke are based on an adaptation of the latest version of the Prevention and Treatment of Pressure Ulcers/Injuries Quick Reference Guide published by the European Pressure Ulcer Advisory Panel, National Pressure Injury, Advisory Panel, and Pan Pacific Pressure Alliance^65^. 

### Recommendations

1. Skin assessment for the risk of pressure injuries over pressure points is recommended in subjects with impaired mobility, sensory perception, older age, and diabetes. (Recommendation I-A);
2. Structured PI risk assessment and PI classification are recommended. (Recommendation I-C);
3. The skin of individuals at risk of PI should be inspected to identify the presence of erythema. (Recommendation I-A);
4. The skin of individuals at risk of PI should be kept clean and appropriately hydrated. (Recommendation I-C);
5. Full-thickness excision of pressure sores, including abnormal skin as well as granulation and necrotic tissues, should be performed. (Recommendation I-B);
6. The following factors should be considered for PI surgery: comorbidities, surgical risk, the individual’s clinical condition, and the likelihood of healing with non-surgical versus surgical interventions. (Recommendation I-C);
7. Airflow mattresses can be considered for stroke patients at risk of developing PI. (Recommendation IIa-B);
8. Pulsed current electrical stimulation to facilitate wound healing in recalcitrant PI should be considered. (Recommendation IIa-A);
9. High absorbency incontinence products can be used to protect the skin in stroke patients with urinary incontinence at risk of PI. (Recommendation IIa-B);
10. Post-stroke individuals at risk of PI can undergo nutritional assessment. (Recommendation IIa-B);
11. Stroke patients with, or at risk of, pressure injuries can be repositioned on an individualized schedule. (Recommendation IIa-B);
12. Hydrogels, hydrocolloids, and polymeric wound dressings for non-infected stage II PI can be considered. (Recommendation IIa-B);
13. Wound dressing with calcium alginate for stages III and IV PI with moderate exudates can be considered. (Recommendation IIa-B);
14. Hydrogel for stage III and IV non-infected PI with minimal exudate is recommended. (Recommendation IIa-B);
15. Subjects at risk of PI may be encouraged to sit out of bed for limited periods. (Recommendation IIb-B);
16. Offering high-calorie, high-protein fortified foods or nutritional supplements in addition to the usual diet might be considered for stroke individuals at risk of PI. (Recommendation IIb-C);
17. The benefits of topical antiseptics that are active against biofilms are uncertain. (Recommendation IIb-C).

## NUTRITIONAL SUPPORT

After a stroke, individuals are susceptible to nutritional changes due to a variety of symptoms and sequelae. The risk factors for nutritional changes after stroke are dysphagia, immobility, impaired cognition, as well as reduced food and macro- and micronutrient intake^66^. Approximately 50% of stroke patients suffer from malnutrition^67^. 

For individuals without dysphagia and who are not malnourished or at risk of malnutrition, the use of oral nutritional supplements is not indicated^66^. Oral supplements are indicated for individuals who are able to eat and have been diagnosed with malnutrition or were at risk of malnutrition during hospital admission^66^.

Individuals with dysphagia who need food texture modification or fluid thickening should be referred to a dietitian to ensure adequate nutrition and water intake^66^. If oral feeding is not possible, feeding by a nasogastric/enteric tube is recommended^67^. Patients with severe dysphagia, probably lasting longer than seven days, should receive early enteral nutrition, preferably in the first 72 hours^67^. If enteral nutrition is needed for a period longer than three weeks, a percutaneous endoscopic gastrostomy is recommended^67^. 

Sarcopenia is a complication of malnutrition after stroke, and it is associated with an increased risk of falls, fractures, functional disability, rehabilitation difficulties, and mortality^68^. Sarcopenia is caused by increased inactivity, muscle atrophy, neural loss, and bed rest^69^ ^,^ ^70^. The most severe muscle loss occurs in the limb affected by the brain injury^71^. The instrument recommended for identifying the risk of developing sarcopenia is the SARC-F (sluggishness, requiring assistance in walking, rising from a chair, climbing stairs, falls) questionnaire^72^. It assesses muscle strength, muscle quantity/quality, and physical performance. 

### Recommendations

1. Screening for the risk of malnutrition is highly recommended within the first 48 hours of hospital admission. (Recommendation I-C);
2. If oral feeding is not possible, feeding by a nasogastric/enteric tube is recommended. (Recommendation I-A);
3. Every patient with dysphagia who needs food texture modification or fluid thickening should be referred for nutritional assessment to ensure adequate nutrition and water intake. (Recommendation I-C);
4. Percutaneous endoscopic gastrostomy is recommended when there has been a need for enteral nutrition for more than three weeks. (Recommendation I-A);
5. Patients should be screened for the risk of sarcopenia using the SARC-F questionnaire. (Recommendation I-C);
6. The use of oral nutritional supplements is probably recommended for individuals who are able to eat and have been diagnosed with malnutrition or were at risk of malnutrition during hospital admission. (Recommendation IIa-C);
7. For patients with severe dysphagia lasting longer than seven days, early enteral nutrition is probably recommended. (Recommendation IIa-C);
8. The use of oral nutritional supplements is not recommended for patients without dysphagia and those who are not malnourished or at risk of malnutrition. (Recommendation III-C).

## MOOD DISORDERS

Post-stroke depressive disorder (PSDD) is defined by the presence of a significantly depressed mood or a marked decrease in interest or pleasure that occurs as a consequence of a stroke^73^. It occurs in approximately 30% of patients in the first five years after stroke^74^. The risk of developing PSDD is proportional to the severity of the stroke^75^, and social, genetic, and epigenetic factors^76^. However, the association with the topography of the stroke is not clear^76^. The presence of PSDD increases the risk of death threefold over a 10-year period, particularly in patients with less social support^77^. PSDD is associated with fewer feelings of guilt and a high risk of suicide, and this should be specifically monitored in younger patients with a history of depressive episodes before the stroke^78^.

Adequate social support is necessary to prevent PSDD^79^ ^,^ ^80^. A systematic review and meta-analysis of low quality showed that prophylactic use of selective serotonin reuptake inhibitors (SSRI) in nondepressed stroke patients for one year may reduce the odds for development of post stroke depression^81^. Non-pharmacological treatment of PSDD involves family support, cognitive behavioral therapy, and lifestyle interventions^79^. Patient education about stroke has a positive effect^79^. Physical exercise training is a potential treatment option for PSDD^79^. Transcranial magnetic stimulation is a promising treatment^82^. Pharmacological treatment with antidepressants, especially SSRIs, has been shown to be effective in improving post-stroke survival and in cases of emotional lability^79^ (Table 1). Neuroleptics, anticonvulsants, and lithium have been used for post-stroke manic symptoms^79^. 

### Recommendations

1. Pharmacological treatment with antidepressants, such as SSRIs, can be recommended for the treatment of PSDD. (Recommendation IIa-A);
2. Selective serotonin reuptake inhibitors may be used prophylactically after stroke. (Recommendation IIb-B);
3. Family support, cognitive behavioral therapy, and lifestyle interventions can be considered. (Recommendation IIa-B);
4. Exercise training may be used as a complementary treatment option in cases of PSDD. (Recommendation IIb-B);
5. The combination of pharmacological and non-pharmacological treatments may be considered. (Recommendation IIb-B);
6. Transcranial magnetic stimulation has unclear benefits. (Recommendation III-B).

## DEEP VEIN THROMBOSIS 

Acute stroke survivors are at high risk of deep vein thrombosis (DVT) and pulmonary thromboembolism (PTE), with incidence ranging from 10% to 75% in this population^83^_._ The main risk factors for post-stroke DVT are advanced age, atrial fibrillation, limb paresis, or plegia^84^. DVT may be present on the second day, with a peak incidence from the second to the seventh day and may persist during the rehabilitation phase in 30% of patients with severe paresis^83^. The main complications of DVT in post-stroke patients are post-thrombotic syndrome and PTE, which can occur in 15% of cases with proximal DVT and account for approximately 3% of post-stroke deaths^85^. 

 Non-pharmacological interventions have been effective in preventing DVT and PTE. The randomized CLOTS 3 study showed that in patients with ischemic or hemorrhagic stroke, intermittent pneumatic compression was effective in reducing DVT and possibly improved survival^86^.

For the prevention of DVT or PTE in patients with ischemic stroke, a meta-analysis^87^ concluded that: 1) intermittent pneumatic compression should be used in immobilized patients; 2) elastic compression stockings are not indicated; 3) prophylactic anticoagulation with unfractionated heparin (UFH)* or low molecular weight heparin (LMWH)** or heparinoids should be considered in immobilized post-stroke patients for whom the benefits of reducing the risk of DVT outweigh the risk of intra- or extracranial bleeding; 4) if anticoagulation is chosen, LMWH or heparinoids should be prioritized over UFH due to the greater reduction in the risk of DVT, better ease of use, cost reduction, and patient comfort; and 5) LMWH is associated with a higher risk of extracranial bleeding, with the risk being higher in elderly patients with renal dysfunction. 

 A double-blind randomized study showed that in critically ill patients, including 15% of patients with ischemic stroke, after hospital discharge, rivaroxaban at a dose of 10 mg/day for 45 days reduced the combined risk of fatal and severe thromboembolism by approximately 28%, without a significant increase in bleeding tendencies^88^. 

 Regarding hemorrhagic stroke, a prophylactic dose of heparin between the second or fourth day did not increase the risk of intracranial bleeding, despite the low quality of the evaluated studies^89^ ^-^ ^91^. 

 In patients with ischemic stroke and DVT or PTE, the anticoagulation maintenance period will be three months, unless another overlapping medical condition increases the risk of recurrence^92^. 

 Literature lacks studies using the inferior vena cava filter (IVCF) in cases of hemorrhagic stroke. However, considering its use in other conditions with contraindications for the use of anticoagulants, the use of inferior vena cava filters in patients with hemorrhagic stroke can be considered^93^ ^,^ ^94^.

 *Recommend dose of unfractionated heparin: < 15.000 UI/day. ** Recommended dose for low molecular weight heparin: 30 to 60 mg/day. 

### Recommendations

1. Intermittent pneumatic compression is recommended in immobilized post-stroke patients to prevent DVT. (Recommendation I-A);
2. In ischemic stroke, prophylactic doses of UFH or LMWH should be used during the hospital stay or even after discharge until the patient regains mobility. (Recommendation I-A);
3. In ischemic stroke, a prophylactic dose of LMWH over UFH can be used to prevent DVT. (Recommendation IIa-A);
4. Rivaroxaban at a dose of 10 mg/day for 45 days can be considered as prophylaxis for thromboembolism. (Recommendation IIa-B);
5. IVCF can be considered in immobilized hemorrhagic stroke patients if anticoagulation is contraindicated. (Recommendation IIa-B);
6. In hemorrhagic stroke, it may be reasonable to use a prophylactic dose of UFH or LMWH to start between the second and fourth days of hospitalization rather than no prophylaxis. (Recommendation IIb-C);
7. The use of elastic compression stockings is not recommended. (Recommendation III-B).

## SECONDARY STROKE PREVENTION 

After an ischemic stroke or a transient ischemic attack (TIA), the risk of recurrence without treatment was 10% in the first week, 15% at one month, and 18% at three months^95^. In the long term, it was 10% in one year, 25% in five years, and 40% in ten years^96^. 

 Meta-analysis of individuals with cardiovascular disease through long-term follow-up identified that a reduction of 1 g/d sodium (2.5 g/d salt) was associated with a decrease in cardiovascular events^97^. Another study established the efficacy of physical activity compared with usual care to reduce risk factors after stroke^98^. Some evidence suggests that smoking cessation and reduced alcohol consumption reduce recurrent events^99^ ^,^ ^100^. 

Antihypertensive therapy reduces the risk of ischemic or hemorrhagic stroke^101^. All classes of antihypertensive drugs have been shown to be equally effective, to the detriment of beta-blockers, due to their permissiveness in pressure variability^101^. The use of statins is recommended regardless of the initial LDL cholesterol level^102^. The target for maximum secondary prevention is an LDL< 70 mg/dl, preferably using high-potency statins, such as rosuvastatin or atorvastatin. 

Prediabetes and diabetes are associated with increased risk of initial ischemic stroke^103^. The American Diabetes Association and European Association for the Study of Diabetes recommend metformin and lifestyle optimization as first-line therapies^104^. To prevent vascular events, including ischemic stroke, GLP-1 receptor agonists should be added^104^.

Antiplatelet agents should be prescribed to patients with non-cardiac-embolic stroke or TIA. Short-term use of aspirin plus clopidogrel for up to 21 days is recommended in patients with acute minor stroke or high-risk TIA^105^. In the long term, agent selection must be individualized based on the risk profile, cost, and tolerance^105^. 

Patients with cardiac embolism, particularly those with atrial fibrillation, should be treated with anticoagulants^105^. Options include warfarin with an adjusted dose INR between 2 and 3 or direct oral anticoagulants (DOACs: apixaban, dabigatran, edoxaban, or rivaroxaban). The safety profile of DOACs is superior to that of warfarin, with equal or superior efficacy in preventing new events^105^.

Patent foramen ovale (PFO) closure is recommended for patients with cryptogenic stroke aged < 60 years, large PFO, or pronounced right-to-left shunt, without other concomitant etiologies^105^.

Severe symptomatic intracranial stenosis or occlusion should be treated with antiplatelet agents, and the combination of clopidogrel with aspirin for 90 days may be reasonable^105^.

The approach to stroke rehabilitation does not differ in the presence of comorbidities. 

### Recommendations

1. Antiplatelet agents are recommended in patients with non-cardioembolic stroke or TIA for secondary stroke prevention. (Recommendation I-A);
2. Anticoagulation with warfarin or DOACs is recommended for stroke or TIA with a cardioembolic source, with a preference for DOACs over warfarin. (Recommendation I-A);
3. Patients with diabetes should control their blood glucose with physical activity, lifestyle modifications, and glucose-lowering agents with proven effectiveness in reducing risk for major cardiovascular events. (Recommendation I-A);
4. In severe symptomatic intracranial stenosis or occlusion, a combination of clopidogrel and aspirin should be used. (Recommendation I-A);
5. An exercise program by a health care professional, in addition to routine rehabilitation, is beneficial for secondary stroke prevention. (Recommendation I-A);
6. Blood pressure control with a goal of systolic pressure less than 140 mmHg and diastolic pressure less than 90 mmHg is recommended. (Recommendation I-A);
7. It is recommended that LDL values be kept below 70 mg/dl. (Recommendation I-A);
8. Quitting smoking and reducing alcohol consumption are recommended. (Recommendation I-B);
9. Reducing sodium intake is recommended to reduce the risk of stroke. (Recommendation II-A);
10. PFO closure is recommended for cryptogenic stroke patients aged < 60 years. (Recommendation IIa-B);
11. For severe symptomatic intracranial stenosis or occlusion, the combination of clopidogrel and aspirin for 90 days should be considered. (Recommendation IIa-B).

## SLEEP DISORDERS 

Post-stroke patients experience insomnia, excessive daytime sleepiness, fatigue, non-restorative sleep, nocturia, and sleep fragmentation, often present even before the stroke^106^. It is important that conventional polysomnography be performed in this population, as stroke can cause respiratory changes that are undetectable by the screening devices available on the market^107^.

Obstructive sleep apnea syndrome (OSAS) affects 50% of stroke patients, and there is a strong interrelationship between the two conditions^108^ ^-^ ^112^. OSAS exacerbates post-stroke deficits by impairing the consolidation of neuroplastic synaptic processes involving cognition and praxis^113^. 

Muscle relaxants, including benzodiazepines, are known to worsen OSAS^106^. Continuous positive airway pressure (CPAP) treatment of OSAS must be performed with a positive pressure sufficient to eliminate the apnea events. The device must be worn in an uninterrupted fashion during sleep, every day of the week, with an appropriate nosepiece^114^. A meta-analysis has shown that the use of CPAP can be beneficial for post-stroke neurological recovery^115^. Whether OSAS treatment also reduces the recurrence of stroke remains controversial^116^.

 Fully-fledged insomnia or symptoms of insomnia affect one-third to nearly half of all post-stroke patients^106^. Antidepressants should be taken in the morning, so avoiding the conditioning effect associated with the idea that night-time use of these drugs is intended to induce sleep^108^. Trazodone has been shown to improve sleep and blood pressure parameters in post-ischemic stroke patients^110^. (Table 1). Cognitive-behavioral therapy is beneficial and positively impacts neurofunctional outcomes^111^.

Excessive daytime sleepiness is frequent in post-stroke patients and is associated with higher mortality and less successful rehabilitation^117^. Restless leg syndrome can have a negative impact on the prognosis of post-stroke patients^118^. 

### Recommendations

1. CPAP is recommended in individuals with post-stroke OSAS. (Recommendation I-A);
2. Excessive daytime sleepiness and restless leg syndrome should be investigated and treated if present. (Recommendation I-B);
3. Trazodone can be considered in individuals with ischemic stroke and OSAS. (Recommendation IIa-A);
4. Conventional polysomnography is probably recommended in individuals with a history of stroke or TIA. (Recommendation IIa-B);
5. Antidepressants should be taken in the morning. (Recommendation IIa-B);
6. Cognitive behavioral therapy can be considered in individuals with post-stroke sleep disorders. (Recommendation IIa-B);
7. Benzodiazepines and muscle relaxants should not be used in the management of post-stroke sleep disorders. (Recommendation III-C).

## FALLS

 Falls are one of the most common causes of post-stroke complications. They can occur in the acute or chronic phases^119^ ^,^ ^120^. Approximately 7% of falls occur in the first week after stroke, 25% to 37% between one and six months, and 40% to 50% at six to 12 months. After one year, falls continue to occur in 73% of patients^121^. Falls are most frequent in the first three weeks of rehabilitation^122^.

Falls are associated with motor, sensory, or visual impairment, cognitive dysfunction, hemineglect, and stroke in the posterior circulation^123^ ^,^ ^124^. The causes of falls include cardiac arrhythmias; orthostatic hypotension; vasovagal syncope; psychological factors, such as depression and fear of falling; seizures; and some drugs, such as antihypertensives, diuretics, anticholinergics, antidepressants, and antiepileptics^124^ ^-^ ^128^.

Prevention of falls can be achieved by supervision, strength training, improvement of balance and cognition, less use of sedative drugs and diuretics, and counseling to avoid risky situations^129^ ^,^ ^130^. Physical activity showed positive outcomes in long-term stroke patients, mainly with specific tasks to improve postural stability, walking in challenging situations, and agility training programs for effective fall prevention^131^ ^-^ ^133^. A systematic review with meta-analysis showed a reduction in falls in post-stroke patients with the practice of ancient tai chi^134^.

### Recommendations

1. Exercises aimed at preventing falls, with training to improve balance, are recommended. (Recommendation I-B);
2. Prevention of falls through patient supervision, reduction of the use of sedatives and diuretics, and restriction of activities with a risk of falling should be instituted. (Recommendation I-C);
3. Tai chi can be considered for fall prevention. (Recommendation IIa-B);
4. Agility training programs for fall prevention is reasonable. (Recommendation IIa-C).

## OSTEOPOROSIS

Osteoporosis is a metabolic bone disease characterized by an imbalance between bone resorption and accumulation, leading to changes in the bone microarchitecture and a reduction in bone mineral density (BMD)^135^ ^,^ ^136^. In addition to spasticity, changes in geometric bone properties on the paretic side, increased skeletal fragility, and accelerated bone loss that occurs after a stroke, result in osteoporosis due to disuse^135^. This loss of bone mass as well as reduction in bone structure is greater on the paretic side than on the non-paretic side and affects the upper limbs more than the lower limbs^135^. The risk of fractures in stroke patients is seven times higher than that in the same population according to sex and age^136^. Eighty percent of fractures occur on the paretic side. 

The evidence for drug treatment strategies for osteoporosis in stroke patients is limited^137^. It is not known who is eligible, the best timing, which drug is better, and the best duration of treatment^138^. Further studies are needed to recommend calcium and vitamin D supplements^139^ ^,^ ^140^. However adequate supplementation of both can be used in all post-stroke patients^141^ ^,^ ^142^. Bisphosphonates such as zoledronic acid are a therapeutic option for both oral and intravenous administration^143^ ^,^ ^144^. Hormonal therapy, tibolone, and selective estrogen receptor modulators have cardiovascular risks^145^.

Some medications, such as warfarin, pioglitazone, enzyme-inducing anticonvulsant drugs^146^ and selective serotonin reuptake inhibitors, are associated with an increased risk of fracture^147^. There are clinical studies showing the potential benefits of statins in preventing osteoporosis and fractures^148^. 

Physical activity with gait training and resistance exercises may have some beneficial effects on BMD loss, but there is limited evidence^149^. 

### Recommendations

1. Vitamin D and calcium supplementation can be recommended for stroke patients. (Recommendation IIa-C);
2. Bisphosphonates can be used. (Recommendation IIa-B);
3. Statins can be beneficial in preventing osteoporosis after stroke. (Recommendation IIa-B);
4. Physical activity with gait training and resistance exercises can be useful. (Recommendation IIa-C);
5. Selective estrogen receptor modulators, warfarin, pioglitazone, enzyme-inducing anticonvulsants, and selective serotonin reuptake inhibitors can be used with caution. (Recommendation IIb-B);
6. Tibolone should be avoided. (Recommendation III-B).

## SEIZURE MANAGEMENT

Stroke is the leading cause of epilepsy among individuals over 60 years of age^150^. The incidence of post-stroke epileptic seizures is 7% and may be higher in cases with cortical involvement, greater severity of the vascular event, and hemorrhagic stroke^151^. Epilepsy is associated with increased mortality, prolonged hospitalization, and higher rates of disability^152^. In stroke patients, the risk of subsequent seizures after an unprovoked seizure is approximately 70%^153^. A single unprovoked seizure is sufficient for the diagnosis of epilepsy. 

The risk of acute symptomatic seizures or unprovoked seizures is low. Even in patients with hemorrhagic stroke and cortical involvement, the risk does not exceed 35%. Therefore, the use of antiseizure medication (ASM) as primary prophylaxis is not justified^154^. Likewise, since the risk of seizure recurrence within seven days of stroke is less than 20%, initiation of ASM after a first symptomatic seizure is generally not recommended^155^. Nevertheless, no adequately powered randomized trial results are available, and this issue is still being debated^156^. In addition, there is a lack of data to determine the differences between ischemic and hemorrhagic stroke-related seizures in terms of risk factors and treatment approaches^150^. Therefore, the guidelines end with generalized recommendations^155^. In practice, clinicians consider the risk of clinical worsening following seizure. It is therefore reasonable to base the decision on stroke severity, injury location, stroke subtypes (intracerebral hemorrhage/subarachnoid hemorrhage), and electroencephalogram findings^150^ ^,^ ^156^. If ASM is used for some reason, it should be limited to the acute phase^155^.

Conversely, the risk of recurrence after an unprovoked seizure is approximately 70%, which defines epilepsy. In this situation, the use of ASM as secondary prophylaxis should be considered. The decision of a possible future suspension of ASM must be individualized since the risk of seizures after ASM withdrawal is high in patients with structural damage^155^.

Most patients with post-stroke epilepsy have seizure control with monotherapy alone^156^. The drugs that have proved to be effective in controlling focal epilepsy are carbamazepine, levetiracetam, phenytoin, and zonisamide for adults, with lamotrigine and gabapentin for the elderly^157^. However, there is no current evidence for ASM choice in stroke patients. The newer ASMs seem to be better tolerated, with fewer drug interactions and better side effect profiles^150^. In a systematic review with network meta-analysis, levetiracetam and lamotrigine were better tolerated than controlled-release carbamazepine for post-stroke epilepsy, with no significant differences in seizure control^157^ (Table 1). 

### Recommendations

1. Long-term use of antiseizure medication after an unprovoked seizure is recommended. (Recommendation I-B);
2. Recurrent post-stroke seizures must be treated, and the selection of antiseizure medication should consider the patient´s characteristics. (Recommendation I-B);
3. Use of antiseizure medication after an acute symptomatic seizure is generally not recommended, but it can be considered during the acute phase. (Recommendation IIa-B);
4. Use of antiseizure medication as primary prophylaxis of post-stroke seizures is not recommended. (Recommendation III-B).

## NEUROGENIC LOWER URINARY TRACT DYSFUNCTION AND FECAL INCONTINENCE 

Post-stroke neurogenic lower urinary tract dysfunction (NLUTD) is defined as a dysfunctional condition of the muscles of the bladder, urethra, urethral sphincter, and pelvic floor, and is related to the topography of the damage caused by the stroke, leading to abnormal or difficult control in voluntary and/or involuntary muscle contraction and/or relaxation during the storage and voiding phases of the bladder^158^.

Approximately one-third of adult stroke survivors have symptoms related to NLUTD^159^ with a prevalence ranging from 11.1% to 70%. Detrusor hyperactivity is the most prevalent symptom (64.7%)^160^ and urinary incontinence is associated with a high risk of death after a new stroke^161^.

Fecal incontinence (FI) is the inability to control bowel movements, causing stool to unexpectedly leak from the rectum. The prevalence of FI is approximately 40% in the post-stroke acute phase and 20% during rehabilitation. The risk factors are age and functional limitations^162^.

Due to the low quality of the studies, no significant effects on NLUTD in post-stroke individuals have been shown by behavioral interventions, assistance from specialized professionals, complementary therapies such as acupuncture (electroacupuncture and moxibustion), transcutaneous electrical stimulation, physical therapy techniques, pharmacotherapy with oxybutynin or estrogen, and a combination of interventions^163^.

There are few studies in the literature on interventions for FI in post-stroke individuals, and they show that educational actions and dietary control have inconclusive effects^164^.

### Recommendations

1. For post-stroke NLUTD, behavioral interventions, specialized professional care, complementary therapies such as acupuncture (electroacupuncture and moxibustion), transcutaneous electrical stimulation, physical therapy techniques, pharmacotherapy, and a combination of interventions have uncertain benefits. (Recommendation IIb-B);
2. For post-stroke FI, educational actions and dietary control have inconclusive effects. (Recommendation IIb-B).

## SEXUAL DYSFUNCTION 

Sexual dysfunction after stroke is underrecognized. It affects over half of stroke survivors and it is not solely attributed to the physical effects of stroke^165^. Fewer than 10% of patients receive any advice, despite 90% of patients hoping for advice relating to sexual dysfunction in stroke. Symptoms are characterized by changes in sexual activity, sexual dissatisfaction, decreased libido, problems in achieving orgasm, and erectile dysfunction (ED)^166^. 

Sexual dysfunction is associated with depression, fear of recurrence of a new stroke, and self-perception of impaired motor function^167^. Antihypertensive drugs, depression, and anxiety are associated with ED^168^.

Sexual rehabilitation involves counseling and non-pharmacological and pharmacological interventions^169^ ^,^ ^170^. Counseling may address sexual performance related to medication issues and comorbid conditions that may affect sexual function. Orientation to reduce anxiety related to sexual problems involves discussions regarding the ideal timing for sexual activity (in the morning when the person is not tired), dealing with bladder and bowel issues, and working around the weakness (physical support with pillows), thus helping stroke survivors and their partners. Pharmacological interventions include phosphodiesterase‐5 inhibitors, intracavernosal injections, and intraurethral suppositories to assist erectile function. Non-pharmacological interventions, such as mechanical devices, lubricating gels, and psycho‐educational interventions, are also components of sexual rehabilitation^169^ ^,^ ^170^. 

The effectiveness of interventions to treat sexual dysfunction is limited. According to a recent meta-analysis, data indicating the benefits or risks of using sertraline to treat premature ejaculation, pelvic floor physiotherapy and sexual rehabilitation to treat sexual dysfunction after stroke are insufficient^171^. 

### Recommendations

1. It is recommended that stroke subjects be asked about their sexual function. (Recommendation I-C);
2. Mood disorders and fears should be addressed in sexual dysfunction after stroke.
3. If ED is present in men after stroke, antihypertensive drug use, anxiety, and depression should be investigated. (Recommendation I-B);
4. The benefits of sertraline in treating premature ejaculation are uncertain. Recommendation IIb-B);
5. The effects of sexual rehabilitation for treating sexual dysfunction after stroke are not well established. (Recommendation IIb-B)

## CONCLUSION

The Brazilian Guideline for Stroke Rehabilitation - Part I presents recommendations on interventions to manage and prevent complications and comorbidities after stroke. However, this guideline is open to criticism for potential issues in the recommendations: 1) the variety of topics covered; 2) the diverse effects of a single intervention in recovery from neurological deficits and disabilities; 3) the low methodological quality of the studies evaluated in systematic reviews and meta-analyses; 4) the personal experience of each professional; and 5) the complexity of the theme of stroke rehabilitation. We hope that Part I of this guideline helps the multidisciplinary team in offering the best care of the most frequent clinical conditions after stroke.
